# Drug Utilization Study of Antidiabetic Drugs in Patients Attending Geriatric Outpatient Department at a Tertiary Care Hospital

**DOI:** 10.7759/cureus.17555

**Published:** 2021-08-30

**Authors:** Abdul Hannan, Shyamal R Sinha, Mohammad Arfat Ganiyani, Manas Pustake

**Affiliations:** 1 Department of Pharmacology, Grant Government Medical College and Sir Jamshedjee Jeejeebhoy Group of Hospitals, Mumbai, IND; 2 Department of Internal Medicine, Grant Government Medical College and Sir Jamshedjee Jeejeebhoy Group of Hospitals, Mumbai, IND

**Keywords:** diabetes, anti-diabetic medications, daily defined dose, prescribed daily dose, pdd/ddd ratio

## Abstract

Introduction

Diabetes has increased in prevalence from 108 million individuals in 1980 to 463 million individuals in 2021. As people's life expectancies have risen, it's become increasingly necessary to be worried about diseases that affect the elderly. To focus and manage these diseases effectively, the illumination of current knowledge about the pattern of anti-diabetic drug utilization in the elderly is important. As a result, it is necessary to evaluate the pattern of anti-diabetic medication use among diabetes patients of the geriatric age group and determine if there is room for improvement in light of current knowledge. With this information, we intend to provide feedback and suggestions for the health care providers. This research aimed to study and analyze the drug utilization of antidiabetic medications in patients attending the geriatric outpatient department.

Methods

The data of 600 patients visiting the geriatric outpatient department from January 1, 2016 to September 30, 2017 were collected from the electronic medical record (EMR) database. The protocol was designed using Strengthening the Reporting of Observational Studies in Epidemiology (STROBE) guidelines. Subjects were grouped according to gender, age, drug combination use, and underlying co-morbidities. Indicators of drug usage and the total number of drugs prescribed and prescription patterns were analyzed. Then, the recorded data were classified according to the anatomical therapeutic chemical (ATC) - daily defined dose (DDD) classification. Prescribed daily dose (PDD) values and PDD/DDD ratio of antidiabetic drugs prescribed to a sample of patients (n=600) were calculated. Cost analysis of the prescribed drugs was analyzed and the cost index for each drug is described.

Results

A total of 600 diabetic patients (286 males) were recruited in the study. In the study, the average age of participants was 69.30±11.34 years. The most common comorbidity associated with diabetes mellitus (DM) was hypertension followed by hypertension along with chronic heart disease. Glibenclamide and pioglitazone (thiazolidenediones) had PDD/DDD ratio equal to 1. The ratios for glimepiride (sulfonylurea), metformin (biguanides), sitagliptin (sodium-glucose cotransporter 2 inhibitor), insulin glargine, insulin lispro, insulin aspart, were 1.85, 1.29, 1.66, 1.63, 1.42, and 1.21, respectively, whereas the premixed insulin had a ratio of 0.83. The average cost per prescription was USD 3.36 and around 87.72% of the cost per prescription was due to the prescribed antidiabetics. Metformin + glibenclamide was the most commonly prescribed combination followed by metformin + glimepiride.

Conclusion

On the whole, the principles of rational prescription were followed in accordance with the different WHO drug usage indicators. Many of the drugs prescribed by generic name were supplied from hospital pharmacy thus reducing the burden to some extent.

## Introduction

Diabetes mellitus (DM) is becoming an important public health problem in developing countries, especially in India. The number of people with diabetes has risen from 108 million in 1980 to 463 million adults in 2021 [1]. Type 2 DM is very common among the elderly [2]. Various classes of anti-diabetic drugs including insulin and oral hypoglycemic agents (OHAs) are currently being used in the treatment of diabetes, which acts by various mechanisms to reduce the blood glucose levels in order to maintain optimal glycemic control. The utilization study of these medications is important in clinical practice because it serves as the foundation for implementing changes to drug dispensing policies at the local and national levels. Irrational drug use can lead to adverse outcomes including an increase in the risk of hypoglycemia, a decline in medication adherence, the risk of drug-drug interactions, all of which can invariably lead to an increased risk of hospitalization, fatality rate, and healthcare costs [3]. Drug Utilization Research (DUR) was defined by the WHO in 1977 as “The study of the marketing, distribution, prescription, and use of drugs in a society, with special emphasis on the resulting medical, social and economic implications” [4]. The main implication of such studies is to promote rational medication usage. Drug utilization studies help in developing strategies to utilize health resources most efficiently, they are particularly needed in a developing economy like India where age-standardized disability-adjusted life-years (DALY) for diabetes is increased by 39.6% since 1990, the largest rise among major non-communicable diseases [5].

World Health Organization (WHO) has projected that diabetes will be the seventh leading cause of death in 2030 in the world [6]. With the enhancement of diagnostic and treatment facilities, with better healthcare facilities and awareness, we now have a growing population of elderly people [7]. As this demographic group expands, the disease burden increases as well, putting an additional strain on an already overloaded healthcare system. Proper evaluation of their problems, correct diagnosis, and suitable treatment are the key factors in reducing this disease burden. This aids in the improvement in the patients' quality of life, which is extremely important.

Without knowledge of how drugs are being prescribed and used, it is difficult to suggest measures to change prescribing habits for the better [8]. It, therefore, becomes important to assess the pattern of the usage of anti-diabetic drugs among the diabetic patients of the geriatric age group and to see to what extent there may be scope for improvement in the light of current knowledge. A previous drug utilization study for OHA done in India was by Sultana et al. in 2010 [8,9]. In their study, the majority of type 2 diabetic patients were treated with multiple antidiabetic drug therapy. The most commonly prescribed antidiabetic drug class was biguanides followed by sulphonylureas, thiazolidinediones, insulin, and alpha-glucosidase inhibitors. They have reported that the metformin was most commonly prescribed monotherapy followed by insulin. They had emphasized the need for patient education for promoting rational use of medications to promote drug adherence. This study has also recommended drug utilization studies should be carried out in a large population and at different locations in India so that the utilization patterns may be compared and diabetes management improved, thus suggesting a need for a longer-term study on a larger sample size [9]. Although many similar studies were done previously, no studies particularly were done in the geriatric population. We plan to use these data to offer feedback and suggestions to healthcare professionals. Thus, this study was designed.

## Materials and methods

Setting

The study was done in Geriatric Outpatient Department, Sir JJ Group of Hospitals, Mumbai, one of the largest government tertiary health centre in Western India.

Study design and ethical considerations

A retrospective drug utilization study was conducted after the approval of the institutional ethics committee (IEC Document number: IEC/Pharm/445/2014). The guidelines for Strengthening the Reporting of Observational Studies in Epidemiology (STROBE) were used in the designing of the protocol and the manuscript [10].

Selection criteria

Inclusion Criteria

Prescriptions of both the sexes of the geriatric population (defined as age >60 years) diagnosed with DM since at least five years and started on either OHA or insulin that were selected for the study.

Exclusion Criteria

Patients with other coexistent causes of hyperglycemia (e.g., Cushing's syndrome, pancreatic cancer, or hormone-secreting tumors) were excluded from the study.

Sample size

Six hundred prescriptions were assessed from medical databases/registries as per WHO standards [8] for performing retrospective drug utilization studies.

Study procedure

The data of patients visiting the geriatric outpatient department (OPD) from January 1, 2016 to September 30, 2017 were collected from the electronic medical record (EMR) database, avoiding Hawthorne's bias, and was documented in a systematic case record form. During the period of the study, the sample frame was set at three prescriptions a day, three days a week. Three prescriptions were chosen as follows: on day one, all three prescriptions were picked at the start of the day; on day 2, three prescriptions were picked in the middle of the day; on day 3, three prescriptions were selected at the end of the day, and so on. In the event of an OPD holiday, the prescriptions for that day were allocated to the next working day. This method was adopted because of three fixed geriatric OPD days per week in our hospital and also to eliminate potential bias.

Data collection and analysis

Data were collected relating to participant demographics and clinical characteristics. The demographic data collected included: medical records department number, gender, age, smoking status, marital status, education level, employment status, income, and occupation. The clinical characteristics data obtained were: length of time since diagnosis with DM, body mass index (BMI), and any relevant medical history or co-morbidities in the records, of any diabetes complications. Prescription details like date, number of drugs, names of individual drugs (generic/brand), any fixed-dose combination (FDC) prescribed, and whether the prescribed drugs were available from the hospital pharmacy, dose, dosage form, dosing schedule, and duration of treatment were all recorded. The medicines that were dispensed by the hospital pharmacy were documented. Those who were not distributed from hospital pharmacies were considered to be purchased from outside pharmacy outlets.

Data analysis

Prescription patterns were assessed as defined by the World Health Organization-International Network of Rational Use of Drugs (WHO-INRUD) drug usage indicators [11]. The prescription medications were categorized using the anatomical therapeutic chemical (ATC) - defined daily dose (DDD) system [12]. The Prescribed Daily Dose (PDD) was derived by averaging the daily dosage of the antidiabetic medicines [13]. The PDD/DDD ratio was then computed by using appropriate values.

Cost analysis

The cost of medications prescribed from the hospital schedule was calculated based on the rate contract available in the hospital drug store. The cost of the drugs prescribed from pharmacies outside the hospital was obtained from the Drug Index (DI): February 2017 [14]. The cost parameters calculated were average total cost per prescription, percentage of average cost due to antidiabetic drugs average cost borne by the hospital, average cost borne by the patient. We estimated the price per 10 tablets/capsules (minimum and maximum, as per DI), average monthly cost (minimum and maximum), which was equal to (PDD/dose per tablet) × price per 10 tablets × 3 and cost index (CI) (maximum price/minimum price) for pharmaceuticals prescribed from outside pharmacies. The cost of each drug was modeled in the USD.

Statistical analysis

The descriptive data were reported in percentages for categorical variables and mean(\pm\)SD for continuous variables. All statistical calculations were done using IBM Statistical Package for the Social Sciences (SPSS) version 24 (IBM Corp., Armonk, NY).

## Results

Demographic characteristics

A total of 600 diabetic patients were recruited in this study, out of which 286 (47.66%) were males and 314 (52.33%) were females. In the study, the average age of the male population was 69.75$\pm$11.08 years and that of the female population was 68.85$\pm$11.34 years. Patients were observed to be mostly in the 61-70 years age range (70%) followed by 71-80 years (24.5%).

Comorbidities associated with DM

**Figure 1:**
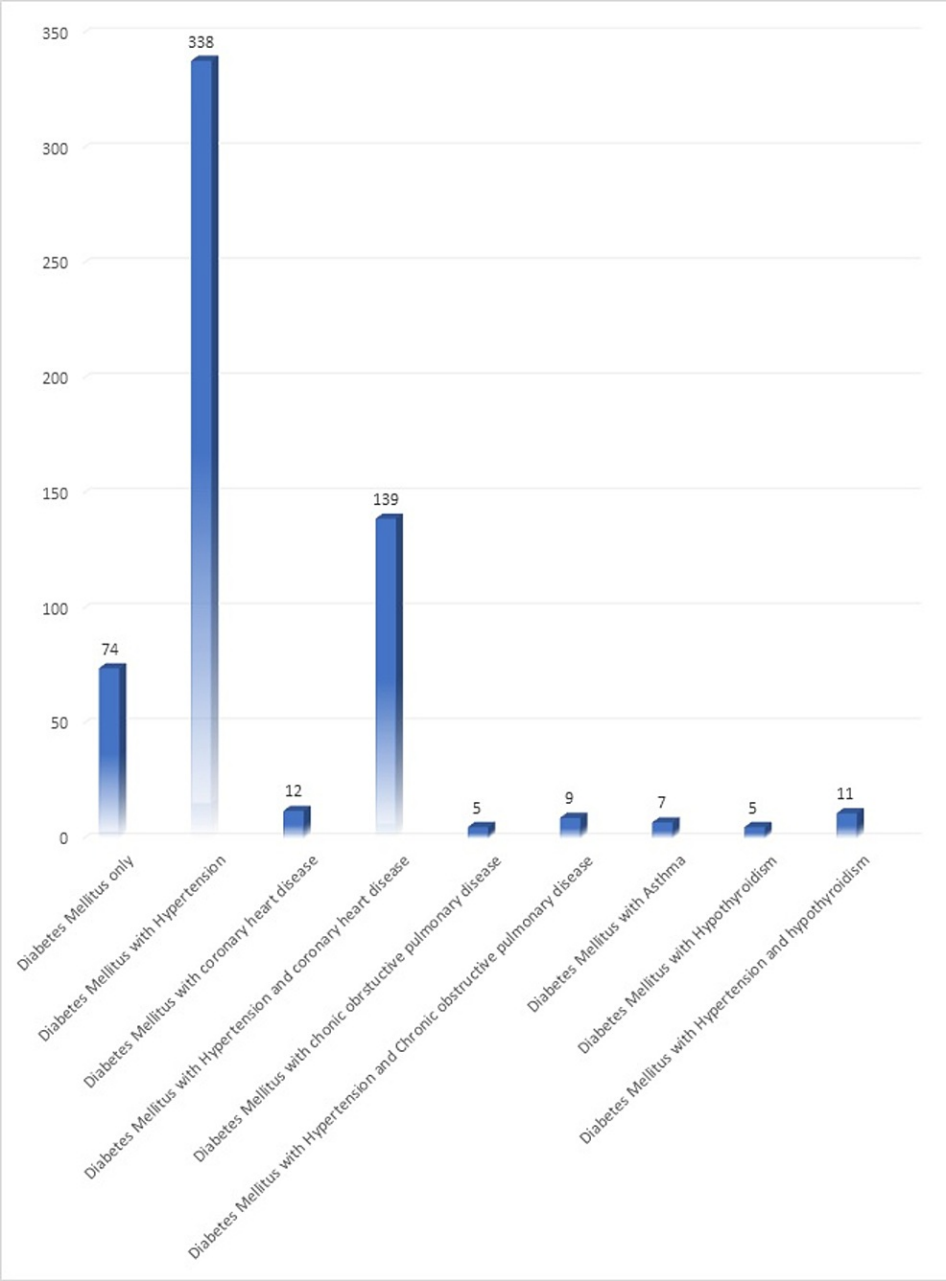
Percentage distribution of diabetes mellitus and co-morbidities (n=600)

The most common comorbidity associated with DM was hypertension (56.33%) followed by hypertension along with chronic heart disease (23.16%); details of which are presented in Figure 1.

Utilization of anti-diabetic drugs

Metformin (biguanide antidiabetic class) was the single most commonly prescribed antidiabetic agent (585 (97.5%) of 600 study participants). It was followed by the sulfonylureas group, which was used by 53.66% of patients. Glibenclamide was the most commonly prescribed sulfonylurea in 263 (43.83%) patients followed by glimepiride in 59 patients (9.83%). The percentage distribution of antidiabetic drugs is shown in Table 1.

**Table 1:** Percentage distribution of antidiabetic drugs in the patients studied DPP-4: dipeptidyl peptidase-4, TZD: thiazolidinedione, NPH: neutral protamine Hagedorn.

Drug class	Drug	Number of patients (%)
Biguanides	Metformin	585 (97.5)
Sulfonylureas	Glibenclamide	263 (43.83)
	Glimepiride	59 (9.83)
Alpha-glucosidase inhibitor	Voglibose	42 (7.0)
DPP-4 inhibitor	Sitagliptin	9 (1.5)
TZD	Pioglitazone	27 (4.5)
Insulin	Premixed insulin (regular insulin + NPH)	32 (5.33)
	Insulin glargine	10 (1.66)
	Insulin aspart	8 (1.33)
	Insulin Lispro	2 (0.33)

**Figure 2:**
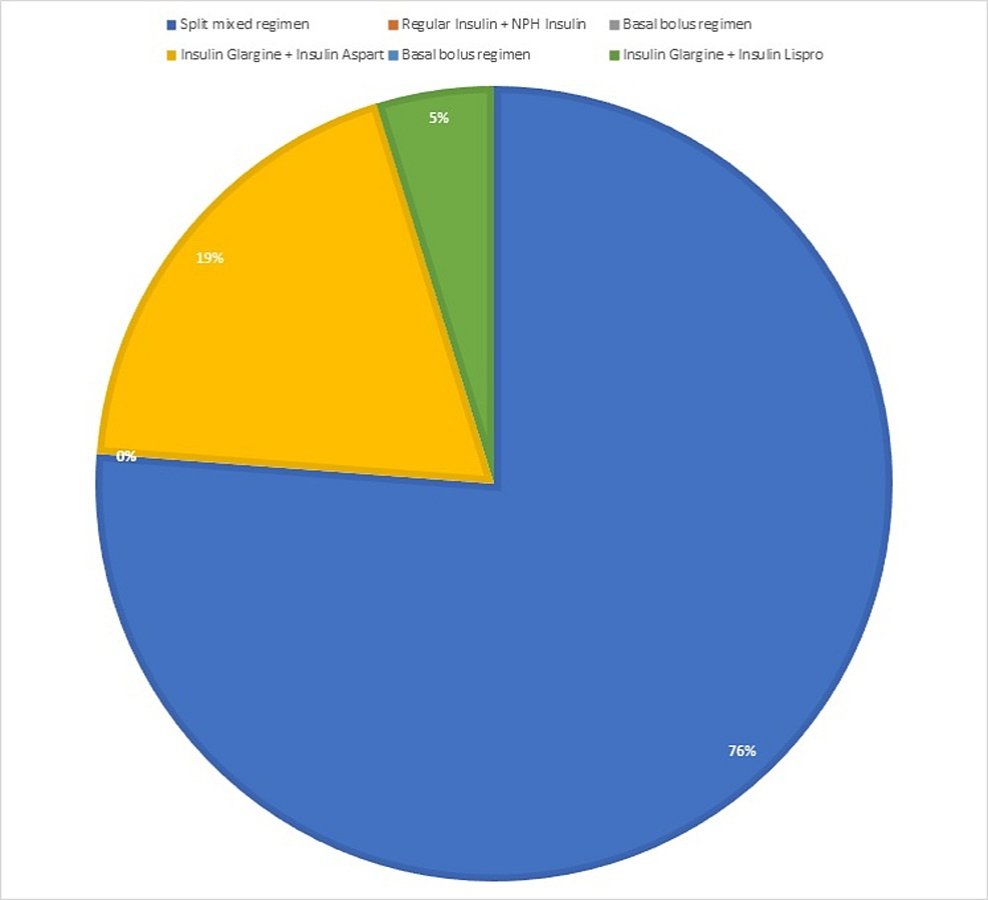
Types and percentage of each insulin regimen prescribed

Insulin was prescribed to 42 patients (7%) out of 600 in three types of regimens. The most often prescribed regimen (regular insulin + NPH insulin) was split mixed in 32 patients (76.19%), followed by the basal-bolus regimen (glargine + aspart) in 8 patients (19.04%). Figure 2 provides a more detailed description.

**Figure 3:**
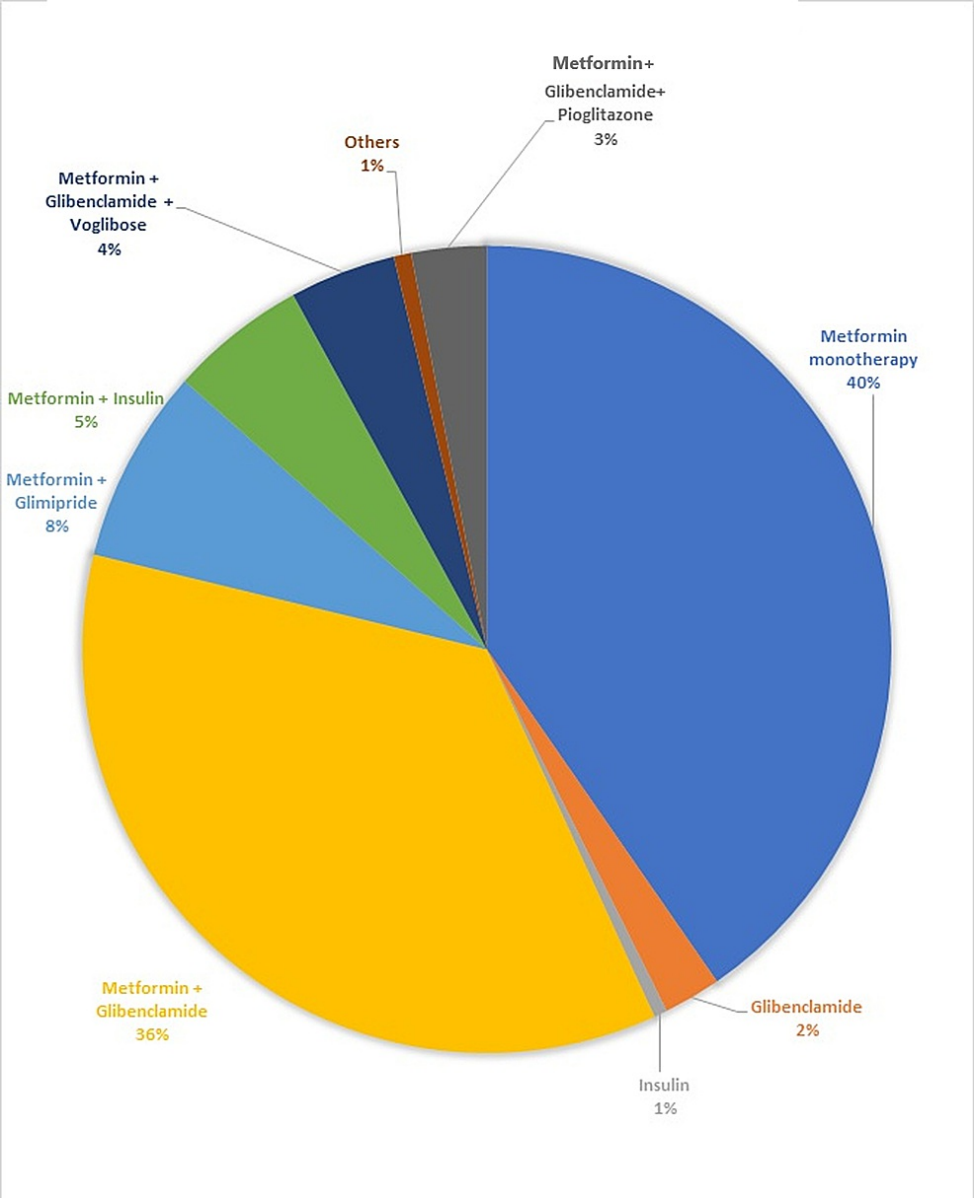
Percentage distribution of antidiabetic drugs prescribed in the study population

The percentage of the study population who were prescribed monotherapy was 40.49, of which metformin in 233 (38%) of patients was the most commonly prescribed drug followed by glibenclamide as shown in Figure 3. Metformin + glibenclamide was the most commonly prescribed combination in 201 (33.5%) of patients followed by metformin + glimepiride in 44 (7.33%) of patients others combinations are described in Figure 3.

**Figure 4:**
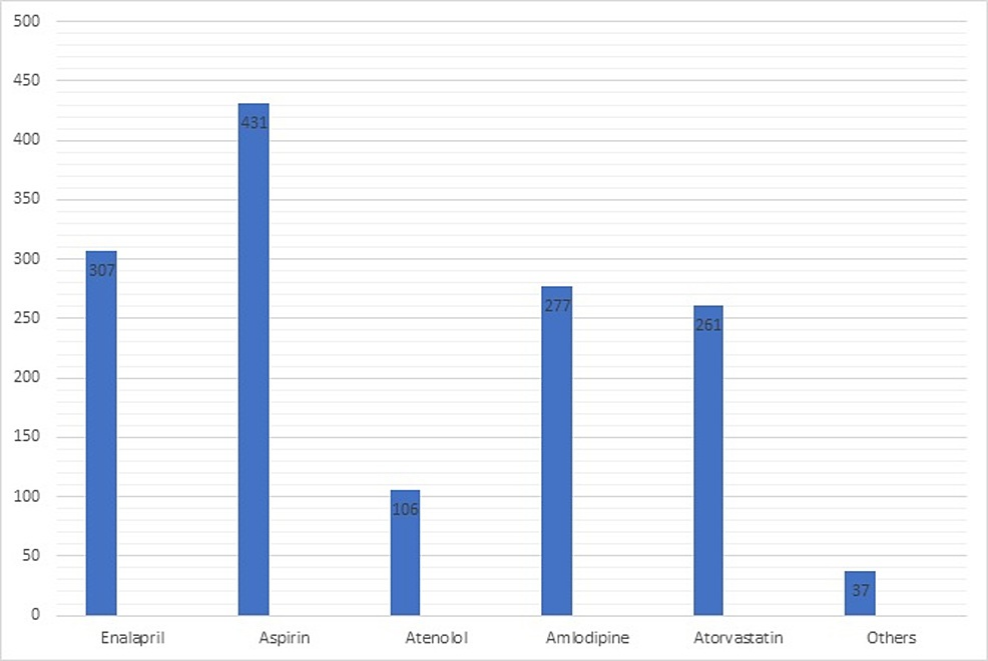
Distribution of drugs in comorbid conditions prescribed along with antidiabetics in patients

The most commonly prescribed drug for the comorbid condition was aspirin in 431 (71.83%) patients followed by enalapril for hypertension in 307 (51.16%) patients. Other drugs and their distributions are elaborated in Figure 4.

Analysis of prescription patterns according to the WHO drug use indicators

Table 2 shows the analysis of prescription patterns according to the WHO drug use indicators.

**Table 2:** Analysis of prescription patterns according to the WHO drug use indicators

Parameter	Findings
The average number of drugs per prescription	4.08 ± 1.31
The percentage of drugs prescribed by generic name	92.68%
The average number of antidiabetic drugs per prescription is	1.71
The percentage of antidiabetic drugs prescribed from the hospital drug schedule	82.57%
The percentage of antidiabetic drugs dispensed from the hospital drug schedule	82.57%
The percentage utilization of scheduled drugs from the National List of Essential Medicines (NLEM) 2015 [15]	65.82%
The percentage utilization of scheduled drugs from the WHO essential list 2017 [16]	56.96%

Drug utilization patterns as per ATC/DDD Classification

ATC/DDD categorization, PDD values, and PDD/DDD ratio of antidiabetic medications are depicted in Table 3.

**Table 3:** ATC/DDD classification, PDD values, and PDD/DDD ratio of antidiabetic drugs prescribed DDD: defined daily dose, PDD: prescribed daily dose, ATC: anatomical therapeutic classification, NPH: neutral protamine Hagedorn.

ATC Code	Drug	DDD	PDD	DDD/PDD
A10BA02	Metformin	2 g	1.54 g	1.29
A10BB01	Glibenclamide	10 mg	10 mg	1
A10BB12	Glimepiride	2 mg	1.08 mg	1.85
A10BF03	Voglibose	Not available on WHO-ATC site	0.44	__
A10BH01	Sitagliptin	0.1 g	0.06 g	1.66
A10BG03	Pioglitazone	30 mg	30 mg	1
A10AD30	Premixed insulin (regular insulin + NPH)	40 U	47.8 U	0.83
A10AE04	Insulin glargine	40 U	24.41 U	1.63
A10AB04	Insulin lispro	40 U	28 U	1.42
A10AB05	Insulin aspart	40 U	33 U	1.21

Cost analysis

The average cost per prescription was USD 3.36 out of which, the cost borne by the hospital was USD 0.81 and that borne by the patient was found to be USD 2.55. Around 87.72% of the cost per prescription was due to the antidiabetics prescribed. Table 4 below summarizes the cost analyses of the drugs.

**Table 4:** Cost analyzes of drugs prescribed to sample of patient attending tertiary care hospital IU: international unit, USD: United States dollar, NPH: neutral protamine Hagedorn.

Drugs	Dose per tablet (mg)	Price per 10 tables/capsules in USD		Average monthly cost USD		Cost index (x/y)
		Minimum (y)	Maximum (x)	Minimum	Maximum	
Metformin	500	0.145	0.337	0.870	2.022	2.32
Glibenclamide	5	0.112	0.385	0.336	1.115	3.43
Glimepiride	1	0.403	0.604	1.209	1.812	1.49
Voglibose	0.3	1.209	2.418	10.881	21.762	2.00
Sitagliptin	500	1.370	3.103	8.220	18.618	2.26
Pioglitazone	15	0.604	0.725	1.812	2.175	1.20
	Dose (IU/mL)	Price per 100 IU in USD				
Premix insulin (regular insulin + NPH)	100	2.471	3.936	37.065	59.04	1.22
Insulin glargine	100	1.961	5.964	5.883	17.892	3.04
Insulin aspart	100	1.873	5.642	6.742	20.311	1.87
Insulin lispro	100	2.418	3.895	5.077	8.179	1.61

## Discussion

Diabetes mellitus (DM) is a rising public health concern in developing countries. Several anti-diabetic drug utilization studies have been published in the healthcare setting from various parts of the world that can aid the rational drug use in patients with diabetes [17,18]. This study has concentrated on trends in anti-diabetic medication prescription and usage. Drug utilization study is important in clinical practice because it serves as the foundation for implementing changes to drug dispensing policies at the local and national levels. Also, since it helps in developing strategies to utilize health resources most efficiently, it is particularly needed in a developing economy like India where 72% of all health care burden is borne by the patients [19].

In our study, we observed that glibenclamide and pioglitazone had a PDD/DDD ratio of 1. Whereas glimepiride, metformin, sitagliptin, insulin glargine, insulin lispro, and insulin aspart had ratios higher than 1 and premixed insulin had a ratio less than 1. When the PDD/DDD ratio is less than or higher than one, it may suggest inadequate use or overuse of drugs, respectively. However, it is important to keep in mind that the PDD may vary depending on the patient and disease variables. PDDs may also vary significantly across nations; for example, PDDs are often lower in Asian people than in Caucasian ones. Additionally, the DDDs acquired from the WHO ATC/DDD website are applicable to moderately severe diseases and are based on worldwide data. As a result, the WHO encourages nations to compile their own DDD list using local data. Our study is contributing to this data, particularly for India.

We found that premixed insulin is underutilized in our settings. In contrast to this, Kalra et al. found in a review that premixed insulin is the most commonly prescribed and used insulin in Asia [20]. This may be attributable to the fact that physicians often have a difficult task in evaluating the contradictory recommendations and deciding which to adopt between basal and premixed insulin [20]. However, the present study justifies the need for prescribing more premixed insulin. Additionally, the present study included individuals in the elderly age range, who may have a preference for non-insulin regimens, resulting in underutilization of premixed insulin.

Pioglitazone is utilized optimally, which may be attributable to a preference for oral hypoglycemic regimes over insulin by patients. It is not overutilized considering its adverse effects.

Metformin alone and metformin combination was the most commonly prescribed anti-diabetic drug in the present study, in line with Orlando and coworkers [21], and Das et al. [22]. They also found that metformin was the most prescribed drug during their study. Interestingly, our results are contrasting to Ramesh and coworkers [23], Chiang et al. [24], and Al Khaja et al. [25], wherein sulfonylureas were the commonly prescribed anti-diabetic drug. This might be attributed to variations in the age groups studied in these studies. In our study, among the second-generation sulfonylureas, glibenclamide was the most commonly prescribed along with metformin which is in line with a study from Nigeria by Jimoh et al. [26]. The fact that metformin was the most prescribed drug complies with its endorsement as the preferred anti-diabetic agent by current clinical guidelines, for instance, the guidelines of the American Diabetic Association (ADA) [27].

This study was conducted in the geriatric department of a tertiary care institution, where the consultants are specialists. In many areas of India, however, diabetes patients are managed by general practitioners. When these physicians are confronted in such situations, the phenomenon of “clinical inertia” is evident. It is referred to as “A consultation in which a change in treatment based on a diabetes-related variable was indicated but did not occur” [28,29]. This leads to inappropriate prescribing and improper use of these medications. To avoid this, government entities must develop and strictly enforce policies. While our research uncovered both the patterns of under- and overuse of anti-diabetic medications, such data may be utilized by government and non-government organizations to develop policies and recommendations to reinforce the appropriate use of these medications. While it is not feasible for countries like India to afford specialists at every level of the healthcare system, it may be mandated for existing healthcare personnel to be trained in order to ensure appropriate medication use, especially for the care of chronic diseases like diabetes.

According to intercontinental marketing service (IMS) statistics, the most often used categories of drugs globally are cardiovascular drugs, which are frequently co-prescribed with anti-diabetic drugs due to the association between cardiovascular illnesses and diabetes [30]. Comorbidities such as hypertension in diabetics make it more difficult to prevent multiple medication usage; as a result, diabetics are more prone to polypharmacy and, in some cases, irrational prescriptions.

While evaluating the rationality, dose strength, and dose schedule that were mentioned in all prescriptions were studied. There was no prescription in which banned drug formulation was prescribed. For instance, pioglitazone which has a black box warning is used in 2.83% of prescriptions which is relatively low. Blood sugar levels were available for all the prescriptions studied. Patients were advised monotherapy as initial therapy and advised dietary restrictions, exercise, and advised eye, cardiovascular, and neurological checkup, which was in adherence with ADA guidelines [27]. This may be attributed to the fact that the study setting is a tertiary care facility, guidelines were followed, which may not be the case in every hospital in the country. Further studies are needed to assess current treatment patterns for good practice and quality of service.

In our study, around 87.72% of the cost per prescription was due to the antidiabetics prescribed. The reason behind this is expensive newer antidiabetic drugs and different preparations of insulin. The costs of diabetes affect everyone, everywhere, but it is not only a financial problem. Unquantifiable costs (pain, inconvenience, anxiety, and overall poorer quality of life, for example) have also been shown to have a significant effect on the lives of patients and their families [31]. It has also been observed that doctors have suboptimal awareness of the costs of the drug. The situation can be improved if drug cost is given greater emphasis during the medical training program of doctors [32]. A mention of drug cost is also required in medical literature and drug advertisement. Either cheaper brands with better efficacy or drugs, in general, should be prescribed as far as possible to reduce the cost of treatment for the patient. In a few instances, pharmaceutical companies use their clout to persuade physicians to prescribe costly medicines, resulting in higher-than-usual prescription costs for consumers.

This study has reported the utilization pattern of antidiabetic drugs in the geriatric population and also provided the baseline data regarding the prescribing patterns in diabetic patients. Since diabetes is a common disorder in the geriatric age group, the prescription cost is one of the major reasons for non-adherence to drug therapy. There is a need to prescribe cheaper alternatives for these types of patients for good glycemic control. This study has opened the door to more research in this field.

Strengths and limitations

Because the study was conducted in a government hospital, there may be a sampling bias since the patients who arrived here are usually from a low socioeconomic class. The population in our study was the elderly age group, hence the actual drug usage by the entire population could not be identified, and these data cannot be extrapolated for the entire population. Additionally, we were unable to evaluate patient compliance since patients were not interviewed in person. A more comprehensive study is warranted. Because of the present study design, we faced certain limitations which could be avoided with another well-designed prospective study.

The study's sample size was adequate. Since we used the EMR, Hawthorne bias was obviated. EMR is a component of the national surveillance system making the gathered data more reliable and accurate.

## Conclusions

On the whole, the principles of rational prescription were followed in accordance with the different WHO drug usage indicators. Many of the drugs prescribed by generic name were supplied from hospital pharmacy thus reducing the financial burden of the patient to some extent. The incidence of poly-pharmacy is relatively high, suggestive of irrational prescribing; but polypharmacy is quite relevant in geriatric diabetic patients because diabetes is associated with various concurrent diseases and their complications. Apart from this, drugs prescribed by generic names were also high, therefore drug use in this setup is quite rational.

We would like to make the following suggestions: (i) the use of premixed insulin was found to be good but further need to increase premixed insulin in hospital drug schedules; (ii) continue the use of metformin adhering to the ADA guidelines; (iii) need to increase newer expensive antidiabetic drugs in the hospital drug schedule and pharmacy in order to relieve the financial recommendation on the patients; (iv) the practice of prescribing glibenclamide and pioglitazone should be continued.
